# Evidence on Sleep and Academic Achievement in First-Year University Students: A Scoping Review

**DOI:** 10.3390/clockssleep8030050

**Published:** 2026-08-24

**Authors:** Diana R. Pereira

**Affiliations:** Human Cognition Lab, Psychology Research Center, School of Psychology, University of Minho, 4710-057 Braga, Portugal; diana.r.pereira@gmail.com

**Keywords:** sleep, academic achievement, first-year university students, higher education, scoping review

## Abstract

The transition from high school to university is a critical developmental period marked by substantial academic, social, and lifestyle changes that may increase vulnerability to academic difficulties, disengagement, and dropout. Given the importance of academic achievement for student retention and long-term success and the growing recognition of sleep as a modifiable determinant of academic performance, this scoping review examined how the relationship between sleep and academic achievement has been investigated among first-year university students. Following PRISMA guidelines, a systematic search identified 20 records representing 21 studies published between 2013 and 2026. Sleep duration and sleep quality were the most frequently examined domains, although findings were heterogeneous. Nevertheless, shorter sleep duration, particularly below 6–7 h per night, was generally associated with poorer academic achievement. Poor sleep quality was also linked to lower academic performance in some studies, with affective factors influencing this relationship. Sleep problems, including chronic sleep deprivation and risk of sleep disorders, showed consistent associations with poorer academic outcomes. Similarly, an evening chronotype was generally associated with poorer academic performance and adjustment. These findings emphasize sleep as an essential target for future research and initiatives aimed at promoting academic success and student retention during the transition to higher education.

## 1. Introduction

The transition from high school to college represents a significant milestone, not only in terms of academic and career development but also at a personal level, as it is often accompanied by multiple familial, social, lifestyle, academic, financial, and developmental opportunities and challenges [1,2]. This transition can be associated with a sense of displacement across multiple domains, as incoming students must learn to navigate new environments (e.g., moving away from the family home, relocating to a different city, or adapting to a new campus), establish new relationships and support networks, manage increased personal responsibilities (e.g., financial obligations, lifestyle changes, scheduling demands, and academic tasks), and cope with negative emotional experiences (e.g., psychological distress, fear, depression, guilt, and shame) [2,3,4]. Although this period offers opportunities for growth and adaptation, it is also characterized by increased vulnerability, as students may encounter difficulties adjusting to university life, including balancing the time devoted to academic demands with the effort required to establish and maintain social relationships [5]. The risk of disengagement and dropout is particularly pronounced during the first year of higher education, underscoring the need for institutions to implement preventive strategies during this critical period and to continually adapt support resources to the evolving needs of incoming students [6].

Among the factors recognized as crucial for promoting student retention, graduation, and long-term academic success, first-year academic achievement has consistently emerged as a key predictor [7,8,9]. Although academic performance is influenced by a broad range of factors (see [10]), modifiable health-related variables constitute particularly important intervention targets. Indeed, the transition to university is often accompanied by substantial changes in health behaviors and lifestyle patterns, representing a period during which new habits related to diet, physical activity, and sleep can be established. These behaviors play a critical role in promoting well-being and resilience while students navigate the daily demands of academic life (e.g., [4,11,12]).

Among these health-related factors, sleep has been identified as a particularly relevant predictor of academic achievement among first-year students [13], with some evidence suggesting that its influence may be comparable to or even greater than that of other health-related behaviors, such as substance use [7]. Research has consistently shown that sleep disturbances and poor sleep hygiene practices are prevalent among first-year university students. These include irregular sleep–wake schedules, prolonged sleep onset latency, using the bed for activities unrelated to sleep, poor sleep quality, and sleep durations below recommended levels (e.g., [14,15,16]). Furthermore, some evidence suggests that poor sleep hygiene behaviors may be more common among college students than among non-clinical adult populations [17]. In this context, it has been argued that interventions targeting sleep may improve academic achievement and, ultimately, contribute to increased student retention rates at universities [7,13,14,18].

Given the importance of understanding first-year students’ sleep experiences and developing tailored interventions to support their adjustment and academic success, the present study aims to review how the relationship between sleep and academic achievement has been investigated in this population and to synthesize the existing evidence across different sleep-related domains.

### 1.1. Sleep Characteristics and Challenges in First-Year University Students

Sleeping experiences among college students are highly heterogeneous, as evidenced by the considerable variability observed across samples and sleep-related measures [15]. Nevertheless, the transition to college is often accompanied by shifts in sleep patterns, including delayed bedtimes and wake times, as well as greater discrepancies between weekday and weekend sleep duration [19,20,21]. Such changes may reflect attempts to compensate for sleep disruptions resulting from the need to balance academic and social demands throughout the week. Current estimates suggest that nearly one in four first-year students sleeps, on average, six hours or less per night [22,23], substantially below the recommended minimum of approximately seven hours of regular sleep per night for young adults and adults [24,25].

Moreover, sleep irregularities may become even more pronounced during periods of heightened academic demands, such as examination preparation and assessment periods (e.g., [26,27]). Poor sleep quality is likewise common among first-year students, with prevalence estimates ranging from 35% to 65% across different student populations (e.g., [28,29,30]). Other sleep-related difficulties, including insomnia symptoms and excessive daytime sleepiness, are also highly prevalent, with reported rates ranging from 18% to 30% for probable insomnia and from 25% to 66% for excessive daytime sleepiness [29,31,32]. Some longitudinal evidence further suggests that these difficulties may worsen over the course of the academic year (e.g., [32]).

The consequences of sleep difficulties are wide-ranging and have been documented across multiple domains. Insufficient or poor-quality sleep has been associated with an increased risk of several adverse health outcomes, including cardiovascular disease, diabetes, obesity, depression, and impaired immune functioning, as well as a greater likelihood of accidents and premature mortality [25]. Among college students, sleep difficulties have been linked not only to health-related and lifestyle behaviors, but also to broader psychosocial and academic challenges. For example, poor sleep has been associated with lower engagement in physical exercise and leisure activities, reduced perceived social support, increased consumption of substances such as alcohol and cannabis, and greater passive screen use (e.g., [20,32,33]). In addition, sleep problems have been related to elevated psychological distress, greater negative affect (e.g., irritability and fatigue), and attentional difficulties, all of which may compromise students’ overall well-being and functioning (e.g., [23,34,35].

### 1.2. Sleep and Academic Achievement

Furthermore, when academic achievement is considered, the influence of sleep on cognition, emotion, and the underlying neurocognitive mechanisms becomes particularly relevant. Although the relationships among these domains have long been recognized in the literature (e.g., [36,37]), the experiences of college students provide a particularly illustrative example of how sleep can negatively affect attention, memory, executive functioning, emotional well-being, and social functioning [18,32,38]. For example, Bermudez et al. [14] proposed a theoretical framework describing a bidirectional and cyclical relationship between sleep difficulties and academic achievement. Specifically, the authors postulated that short sleep duration and poor sleep quality lead to reduced prefrontal regulation, impaired memory consolidation, and increased stress reactivity, ultimately exerting a negative impact on learning and academic performance. In turn, these adverse academic outcomes may trigger feelings of worry and rumination that undermine engagement and resilience, thereby reinforcing sleep difficulties.

Consistent with this framework, available evidence indicates that sleep deprivation impairs memory for information acquired both before and after a period of deprivation, with these adverse effects persisting even when memory retrieval is evaluated after several nights of recovery sleep [39]. Although sleep deprivation and sleep restriction appear to have similar effects on information encoded prior to the period of sleep disruption, memory impairments tend to be more pronounced following sleep deprivation when new information must be learned and encoded [40]. Sleep restriction has also been associated with poorer performance on tasks requiring attention and inhibitory control (e.g., [26,41]). Indeed, it has been suggested that sleep deprivation disrupts functional interactions between prefrontal regions (e.g., dorsolateral and medial prefrontal cortex) and medial temporal structures (e.g., hippocampus, amygdala), resulting in difficulties integrating information from memory- and emotion-related systems, regulating emotions, and exerting inhibitory control over cognitive and affective responses [41,42]. Self-regulatory processes are particularly relevant to understanding the relationship between sleep and academic performance, as students with lower levels of self-regulation and self-control appear to be more susceptible to the detrimental effects of sleep difficulties on academic outcomes than students with stronger self-regulatory abilities [43].

Sleep-related alterations in the connectivity of these neural systems may also contribute to a state of hyperarousal, a phenomenon commonly observed in individuals with mood disorders [42]. This observation is consistent with evidence showing that, among college students, both subjective sleep difficulties and objectively measured sleep duration are associated with anxiety symptoms, somatic complaints, and externalizing behaviors (e.g., reduced adherence to rules) [44,45]. Given that students are regularly exposed to academic and personal stressors, a state of hyperarousal may increase vulnerability to stress, worry, and rumination, thereby contributing to the persistence of sleep difficulties over time [20].

Notably, the bidirectional nature of the relationships proposed by Bermudez et al. [14] is also supported by empirical findings. For instance, difficulties in self-regulated behavior may contribute to the development of sleep problems, while sleep disturbances may further impair the cognitive and emotional processes required for effective self-regulation [43]. Similarly, poor intrapersonal adjustment may increase vulnerability to sleep difficulties, which, in turn, may perpetuate patterns of maladjustment and psychological distress [42].

### 1.3. The Present Study

Based on the evidence reviewed thus far, the role of sleep in academic performance among university students is undeniable. This relationship is further supported by previous systematic reviews, which indicate that inadequate sleep is associated with poorer academic achievement (e.g., [46,47,48]). These reviews also suggest that while some sleep-related domains show relatively consistent associations with academic outcomes, findings for other domains are more mixed. Specifically, poor sleep quality, excessive daytime sleepiness [47,48], and an elevated risk of sleep disorders such as insomnia [46] have consistently been linked to lower academic achievement. Similarly, students who maintain more regular sleep schedules and exhibit a stronger morning chronotype tend to achieve better academic outcomes [46,48]. In contrast, evidence regarding sleep duration remains inconclusive, with some studies reporting significant associations with academic performance and others finding no such relationship [47,48]. However, these reviews encompass students at different stages of their university education. To our knowledge, no comprehensive overview has specifically focused on first-year students, who face unique challenges during the transition to higher education [2] and are at heightened risk of academic difficulties, disengagement, and dropout [6].

Against this backdrop, a more focused examination of the relationship between sleep and academic achievement in first-year university students offers an important opportunity not only to map the existing evidence but also to identify sleep-related targets that could be prioritized in early screening, monitoring, and intervention efforts. Sleep difficulties are a common concern among university students [27], a reality further reflected in students’ reported willingness to miss classes, particularly morning classes, to compensate for insufficient sleep [49]. This apparent awareness of sleep-related challenges may also provide an entry point for addressing other, often more stigmatized, issues closely linked to sleep, such as mental health difficulties [35,50]. Indeed, promoting healthy sleep behaviors may help reduce the likelihood of experiencing poor intrapersonal adjustment during the university years (e.g., [51]).

The primary aim of the present review is to provide a comprehensive overview of how the relationship between sleep and academic achievement has been investigated among first-year university students. A scoping review methodology is particularly well suited to this objective, as it enables the mapping of research trends and methodological approaches while also identifying knowledge gaps within the existing literature [52,53]. Specifically, this review seeks to: (i) characterize how sleep-related variables have been investigated in relation to academic achievement; (ii) synthesize the available evidence across different domains of sleep and identify factors that may influence these associations; and (iii) formulate recommendations for future research, as well as implications for surveillance and intervention initiatives led by stakeholders across the university community with the goal of promoting first-year student success and retention.

## 2. Methods

The Preferred Reporting Items for Systematic Reviews and Meta-Analyses 2020 (PRISMA 2020) guidelines [54], together with the PRISMA extension for Scoping Reviews [55], were used to guide the conduct of this scoping review.

### 2.1. Literature Search Strategy

The identification of potential studies was carried out through searches in three electronic databases: PubMed, Scopus, and Web of Science. Records from database inception to 11 August 2025 were considered. The search strategy included terms related to first-year college students (e.g., first year, freshmen, college, university), academic achievement (e.g., achievement, adjustment, integration, retention, success), and sleep. The full search expressions are provided in Table S1 of the Supplementary Materials. In addition, a supplementary search was conducted by screening the reference lists of studies included in the full-text review and by checking upcoming publications from August 2025 onward to identify any relevant records that may have been missed in the initial database search.

### 2.2. Eligibility Criteria

The eligibility criteria were informed by the Population, Concept, and Context approach (see [52] for a summary). Regarding the population, studies were required to include first-year university students and report data specifically for this group. For the concept, we focused on studies examining academic achievement, adjustment, or engagement (e.g., course grades, adaptation to college) in relation to sleep-related variables (e.g., sleep quality, sleep duration, circadian preference, sleep–wake regularity, sleep onset latency, sleep problems). Regarding the context, we included studies conducted within tertiary education institutions or settings. No language restrictions were applied, and automatic translation tools were used where necessary. Records that did not report original, complete empirical studies, such as conference proceedings, conference abstracts, book chapters, study protocols, systematic reviews, and meta-analyses, were excluded. However, other types of records, including dissertations and preprints, were also considered for eligibility.

### 2.3. Study Selection Procedure

**Figure 1:**
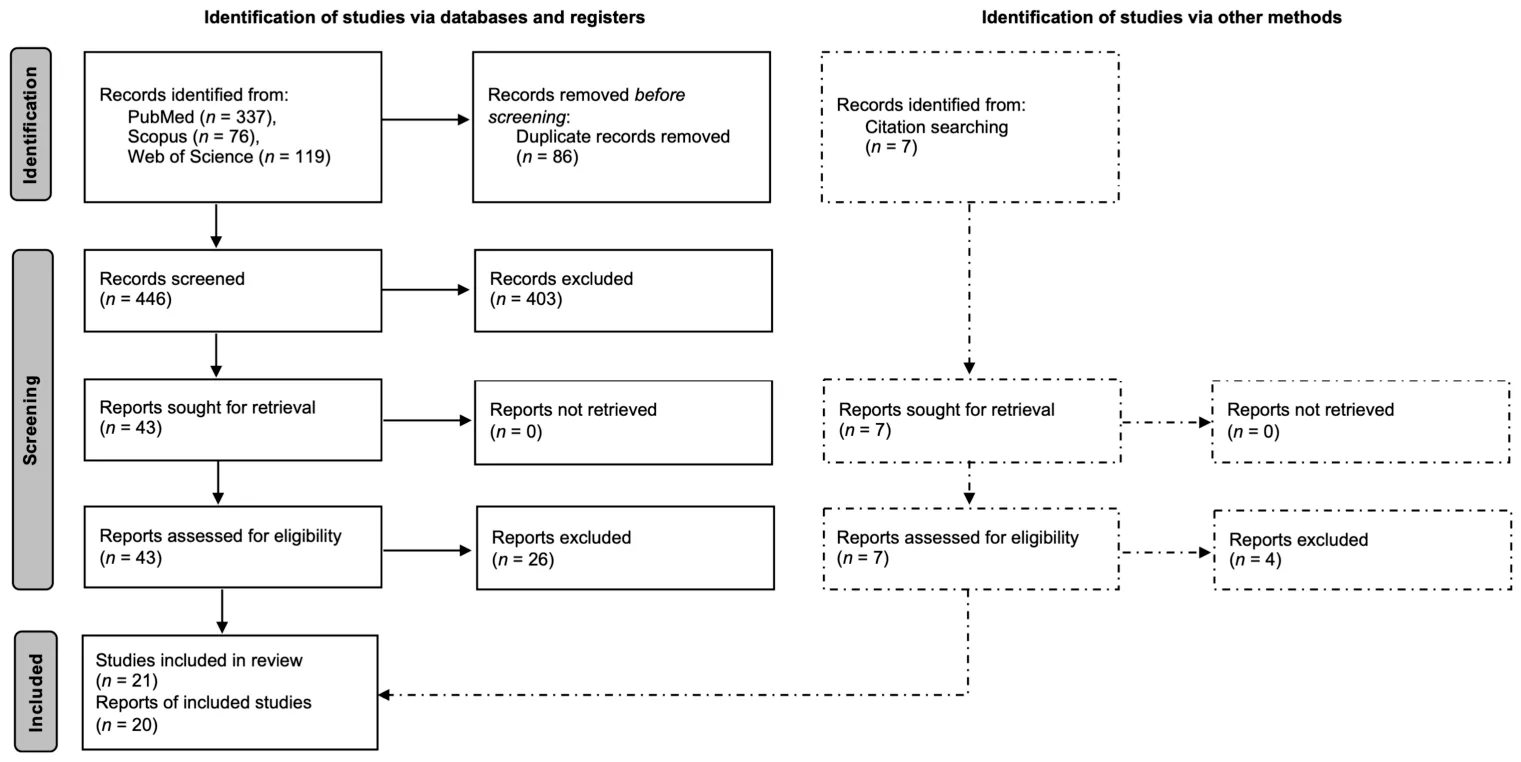
PRISMA 2020 flow diagram depicting the records identified through database searches and other sources and the studies included in the scoping review.

After removing duplicates, the records obtained from the database and citation searches were assessed in two stages according to the eligibility criteria. The first stage involved screening titles and abstracts, followed by a second stage consisting of full-text review. Both steps were conducted by the first author. A summary of the study selection process adopted in this scoping review is presented in the PRISMA flow diagram (see Figure 1).

### 2.4. Data Extraction and Synthesis

A narrative synthesis approach was adopted to organize and interpret the results of this review, as we anticipated a restricted number of studies examining the association between sleep and academic achievement among first-year university students, as well as substantial methodological heterogeneity and variability in findings (see [46,47,48] for reviews). Although such diversity poses challenges for systematic comparison and quantitative synthesis, it also offers an opportunity to reflect on how the field has evolved, which methodological approaches have been employed, and which directions may be most promising for future research. Accordingly, a scoping review supported by narrative synthesis was deemed the most appropriate methodological approach [52,53].

For each study included in the review, we summarized information on study identification (first author’s last name, year of publication, and the country or countries in which the participants were located), participant sociodemographic characteristics (sample size, number of female participants, mean age or age range when means were not provided), the measures used to assess sleep and academic achievement, and the main findings concerning the relationship between these variables (see Table 1).

## 3. Results and Discussion

Following the PRISMA guidelines (see Figure 1), a total of 20 records, representing 21 studies, met the eligibility criteria and were included in this review. The main reasons for excluding reports assessed for eligibility were not reporting findings for first-year university students, not including achievement- or sleep-related measures, or not exploring the association between sleep and academic achievement. Table 1 provides a summary of the main characteristics of the included studies.

**Figure 2:**
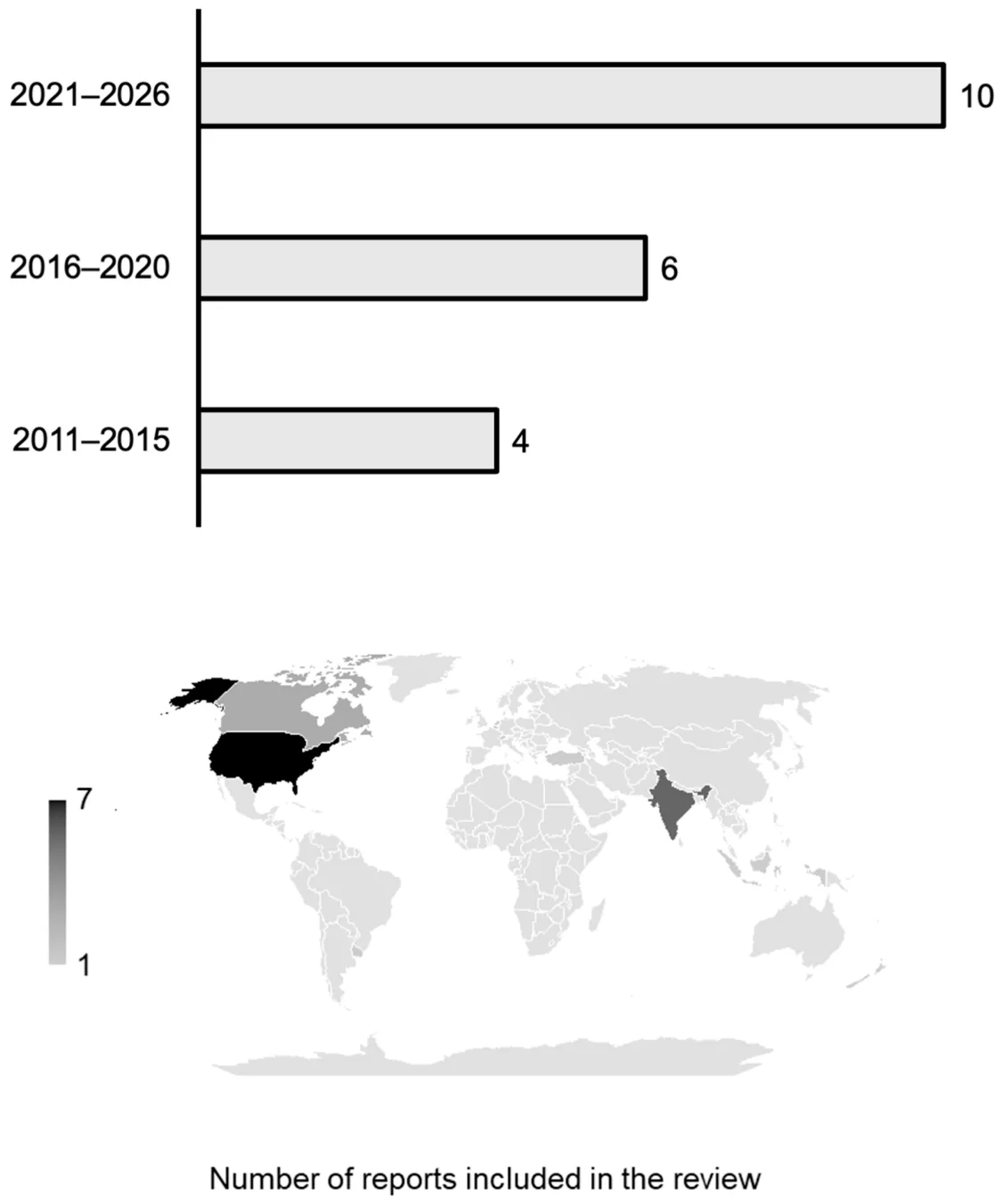
Number of records included in the review by publication period (2011–2015, 2016–2020, 2021–2026) and their geographical distribution.

Research on this topic is relatively recent, with the earliest included record published in 2013. Nevertheless, interest in the transition to college as a critical period for examining the relationship between sleep and academic achievement has grown over time. Notably, half of the included records (n = 10; corresponding to 11 studies) were published within the last five years (see Figure 2).

Most studies were conducted in the United States (n = 7), followed by India (n = 4) and Canada (n = 2). Additional studies were carried out in Belgium (n = 1), Indonesia (n = 1), the Netherlands (n = 1), New Zealand (n = 1), Switzerland (n = 1), Turkey (n = 1), and Uruguay (n = 1). Of the ten countries represented, the majority were high-income countries (n = 7), followed by upper-middle-income countries (n = 2), with only one lower-middle-income country represented (n = 1). Furthermore, seven of the ten countries could be classified as Western, Educated, Industrialized, Rich, and Democratic (WEIRD), highlighting a predominance of evidence derived from these sociocultural contexts.

In the following subsections, we provide an overview of participant characteristics, methodological approaches, and key findings, organized according to the sleep domains examined: sleep duration and timing, sleep quality, sleep problems, and chronotype (see Table 1).

### 3.1. Participants

Sample sizes ranged from 37 participants [58] to 15,090 participants [7], with an average sample size of 1523 participants. Based on tertile distribution, seven studies were classified as having small samples (≤148 participants), six as having medium-sized samples (167–499 participants), and eight as having large samples (≥634 participants). The number of female participants, reported in 18 studies, ranged from 17 [58] to 3721 [14]. Mean age was available for 14 studies and averaged 19.58 years (SD = 1.88), indicating that the samples were largely representative of the traditional first-year university population. Most studies (n = 9) included students from a broad range of academic disciplines. Among the remaining studies, seven focused on students enrolled in health-related programs, including five conducted exclusively with medical students. Three studies examined students pursuing STEM degrees, while two focused on psychology students.

### 3.2. Methodological Characteristics

The 21 studies included in this review exhibited a relatively balanced distribution of study designs, comprising 11 cross-sectional studies (52.4%) and 10 longitudinal studies (47.6%). A closer examination of the longitudinal studies revealed two distinct approaches. Five studies included at least two assessment time points during the first year of university, whereas the remaining five collected an initial assessment during the first year and subsequently followed participants across later stages of their academic trajectory.

Regarding sleep assessment, the predominant approach relied on subjective measures (n = 20). These included self-report items assessing sleep duration (e.g., [12,14,62], sleep-related problems (e.g., [43,57]), daytime sleepiness (e.g., [43]), circadian preference (e.g., [65]), and other sleep-related constructs. Several studies also employed sleep diaries (e.g., [26,29]) and validated instruments, including the Pittsburgh Sleep Quality Index (PSQI), Epworth Sleepiness Scale (ESS), Morningness–Eveningness Questionnaire (MEQ), Sleep-50, Munich Chronotype Questionnaire (MCTQ), and Insomnia Severity Index (ISI). Among these instruments, the PSQI was the most frequently used questionnaire (n = 7), including one study that utilized only selected items from the scale. The ESS (n = 3) and the MEQ (n = 2) were the next most commonly employed measures. Notably, only one study adopted an objective assessment approach by incorporating actigraphy-based measures of sleep [22].

With regard to academic achievement, Grade Point Average (GPA) and related indicators, such as course grades and examination performance, represented the most commonly used outcomes. Nevertheless, several studies extended their analyses to other indicators of academic success and adjustment, including credits earned [43], dropout rates [13,43], graduation outcomes [57], prior academic achievement [13], validated measures of adaptation to university life (e.g., the Student Adaptation to College Questionnaire [SACQ]) [65], and single-item indicators, such as perceived achievement of learning goals [61].

### 3.3. Synthesis of Findings

#### 3.3.1. Sleep Duration and Timing

According to the recommendations of the National Sleep Foundation [24], the sleep duration for young adults is expected to be between 7 and 9 h per night. Indeed, when considering the relationship between sleep duration and academic achievement, sleeping less than 7 h appears to have a deleterious effect on academic performance [14,26], with fewer than 6 h of sleep being particularly detrimental [22,26]. Shorter sleep durations have also emerged as predictors of end-of-year GPA [13], and this appears to be the case even when early-term sleep duration is considered [22]. This association seems to persist even after controlling for a wide range of sociodemographic (e.g., gender, age, race/ethnicity, first-generation college student status), academic (e.g., major, high school academic index, academic load), health and lifestyle (e.g., diet, screen time), and psychosocial factors (e.g., perceived stress, anxiety) (e.g., [13,14,22,26]).

Furthermore, studies exploring the association between sleep duration prior to a specific assessment and performance on that assessment suggest that not only is a greater mean sleep duration during the week preceding the assessment associated with better grades [29], but also that even a one-hour increase in regular sleep duration can improve performance accuracy [60]. When focusing on the nights preceding an evaluation, during which some students may adopt a cramming strategy [67], and considering that sleep duration may decrease as the exam day approaches [68], findings are mixed. Some studies indicate that students who sleep the recommended number of hours are more likely to obtain a passing grade than those who pull all-nighters, and that an additional hour of regular sleep before an assessment may increase the likelihood of answering questions correctly (see survey 1 from [60]). However, other studies have found no association between the amount of sleep obtained prior to an exam and academic achievement [29].

Although the evidence presented thus far appears to support the idea that longer sleep duration is associated with better academic achievement [58], an important question concerns what happens when students sleep more than the recommended number of hours. In this context, Bermudez et al. [14] showed that the relationship between sleep duration and academic achievement is not linear, as sleeping more than 9 h may also predict poorer academic performance.

It is worth noting, however, that some studies have documented no significant relationship between sleep duration and academic achievement [12,27,28,66] or performance on specific assessments (see survey 2 from [60]). Specifically, in the study by Sutay et al. [27] involving first-year medical students, the association between sleep duration and academic performance emerged only for academic performance prior to entering college. Moreover, this association was non-linear, with students sleeping 6–7 h demonstrating the highest levels of performance, even compared with those whose sleep duration was more closely aligned with the recommendations (i.e., 7–8 h). Similarly, in the longitudinal study conducted by Tavernier and Willoughby [66], neither weekday nor weekend sleep duration emerged as a significant predictor of academic achievement over time.

#### 3.3.2. Sleep Quality

Similar to sleep duration, findings regarding sleep quality have also been quite variable. This variability has been observed despite the relatively consistent measurement of sleep quality across studies, with the Pittsburgh Sleep Quality Index (PSQI) being a commonly used instrument. On the one hand, better perceived sleep quality appears to be associated with higher academic achievement [64] and greater learning goal attainment [61]. In addition, sleep quality has emerged as an independent predictor of end-of-first-year GPA, even after adjusting for various sociodemographic (e.g., sex), mental health (e.g., anxiety), and cognitive factors (e.g., fluid intelligence) [13]. Furthermore, on days characterized by higher sleep quality, first-year students tend to report greater learning goal achievement [61]. Students reporting poor sleep quality, as measured by the PSQI (total score ≥ 5), also appear to display lower academic achievement than students whose PSQI scores fall within the expected range [29,64]. On the other hand, some studies have emphasized the absence of a significant relationship between sleep quality and academic achievement [28,56] and have reported no differences in academic achievement among students with varying levels of sleep quality (i.e., poor, moderate, and good) [28]. In the longitudinal study conducted by Tavernier and Willoughby [66], sleep quality did not predict academic achievement over time. Douris et al. [58] even reported that students with poorer sleep quality were actually more likely to achieve a higher GPA.

Several factors may contribute to this variability in findings. In particular, mood-related variables may play an important role, as the presence of depressive symptoms appears to moderate the relationship between sleep quality and academic achievement. Specifically, poor sleep quality seems to be particularly detrimental among students experiencing higher levels of depressive symptomatology [13]. Indeed, Flueckiger et al. [61] found that, on days characterized by higher sleep quality, participants reported greater positive affect and lower negative affect, which, in turn, contributed to better perceived learning goal achievement. Moreover, positive affect partially mediated the relationship between sleep quality and learning goal achievement, suggesting that sleep quality was associated with better learning goal achievement partly because it promoted more positive emotional states.

#### 3.3.3. Sleep Problems

The experience of sleep-related problems among first-year students has emerged as a consistent indicator of lower academic achievement across a range of outcome measures, including GPA, risk of dropping out, likelihood of college graduation, probability of receiving an incomplete grade in a course, and the number of study credits earned [7,43,57,62]. When considering studies that have examined specific sleep-related issues, chronic sleep deprivation [57] and being at risk for a sleep disorder [62] were both associated with lower academic achievement. The picture is less clear, however, in the case of daytime sleepiness. Only one study found that students experiencing excessive daytime sleepiness had lower mean GPAs [29], whereas most of the available evidence suggests no significant association between daytime sleepiness and academic achievement [13,43,64].

The relationship between sleep-related problems and academic achievement appears to persist even after controlling for a range of sociodemographic factors, health-related and lifestyle behaviors, and college experiences [7,43,57,62]. Furthermore, after accounting for relevant background and health-related factors, students at risk for a sleep disorder tend to exhibit lower GPAs, particularly during the first two years of university, suggesting that the impact of sleep-related risk on academic performance may be especially pronounced during the early stages of higher education [62]. Sleep problems also appear to be important predictors of poor academic performance, with effects that are comparable to, or even stronger than, those associated with other health-related behaviors, such as marijuana use and binge drinking [7].

Regarding potential explanatory variables, Gaultney [62] found that students who were at risk for a sleep disorder tended to achieve lower GPAs by the end of their first year, not solely because of their sleep difficulties, but also because these difficulties were associated with lower levels of self-efficacy. This relationship remained significant even after controlling for depressive symptoms. These findings suggest that sleep-related risk may undermine students’ confidence in their academic abilities, which, in turn, negatively affects academic performance. Similarly, the association between sleep problems and academic performance appears to be particularly pronounced among students who report lower levels of self-control and self-regulation [43].

#### 3.3.4. Chronotype

Although studies examining the association between chronotype, circadian preference, and academic achievement are relatively fewer in number compared to those focusing on other sleep-related domains, the available evidence appears to support the notion that an evening chronotype and a preference for eveningness are associated with poorer academic achievement [62] and reduced academic adjustment [65]. Moreover, the predictive effect of an evening chronotype on academic achievement remains evident even after controlling for background characteristics, health-related factors, and fluid intelligence [13]. Conversely, students with a morning chronotype tend to demonstrate higher levels of academic achievement than their evening-type counterparts [63]. It is important to note, however, that Gupta et al. [29] found no association between academic achievement, as measured by GPA, and circadian preference, as measured by the MEQ, which assesses an individual’s preference for functioning earlier or later in the day regardless of their actual schedules.

### 3.4. Discussion

The transition from high school to university is a critical period characterized by substantial academic, social, and lifestyle changes, placing first-year students at increased risk of academic difficulties, disengagement, and dropout [5,6]. Among the various factors that may influence first-year students’ risk of dropout, retention, and eventual graduation, academic achievement is consistently identified as a key determinant (e.g., [9,69]). In turn, sleep has emerged as an important factor associated with academic achievement (e.g., [7,13]), while also being susceptible to changes during the adaptation process to university life (e.g., [14,15,16]). Against this backdrop, the present study aimed to examine how the relationship between sleep and academic achievement has been investigated among first-year university students.

The findings of this scoping review indicate that sleep duration and sleep quality are the most frequently studied sleep domains in relation to academic achievement. For both domains, the evidence remains heterogeneous. Several studies reported that shorter sleep duration, particularly fewer than 6–7 h per night (e.g., [14,22,26]), and poorer sleep quality (e.g., [29,64]) were associated with lower academic achievement. Conversely, other studies failed to identify significant associations between these variables (e.g., [28,56]). This inconsistent pattern has also been reported in previous systematic reviews, particularly regarding sleep duration, although to a lesser extent for sleep quality [47,48]. From a methodological perspective, the studies examining sleep duration displayed substantial variation in measurement approaches, which may partly explain the heterogeneity of findings. For example, some studies employed self-report questions in different formats (e.g., [12,14,62]), others used sleep diaries (e.g., [26,29]), and one adopted an objective approach using actigraphy [22]. In contrast, studies focusing on sleep quality were more consistent in their use of validated instruments, particularly the PSQI (e.g., [13,28,29,56,58,64], which assesses participants’ sleep experiences over the previous month. Conflicting findings appeared to be particularly pronounced among cross-sectional studies, suggesting that the timing and method of data collection may influence the observed associations (see Table 1). For example, Estevan et al. [60] conducted two cross-sectional studies and reported that a one-hour increase in both habitual sleep duration and sleep duration on the night preceding an examination was associated with a greater likelihood of answering questions correctly in a midterm test in one sample. However, in a second sample, a similar association was observed only for sleep duration on the night before the test. No association emerged between habitual sleep duration and test performance despite the use of comparable self-report measures, highlighting how variations in sample characteristics and contextual factors may contribute to inconsistent findings.

Furthermore, the inverted U-shaped relationship between sleep duration and academic achievement documented in one of the included studies [14], and supported by prior evidence from school-aged children and adolescents showing that both insufficient and excessive sleep durations are associated with poorer academic outcomes [70], may partly explain the mixed findings observed across studies. Specifically, the detection of significant associations may depend on factors such as the distribution of sleep duration within a sample and the analytical approaches adopted. Interestingly, a potential non-linear relationship has also been suggested for sleep quality among college students [71].

With respect to other sleep-related domains, particularly sleep problems, the evidence was generally more consistent and aligned with previous reviews focused on university students (e.g., [46,48]). Specifically, first-year students experiencing sleep difficulties tended to exhibit poorer academic achievement [7,43,57,62], particularly those at risk of sleep disorders [62] and those experiencing chronic sleep deprivation [57]. One possible explanation is that sleep difficulties may be associated with alterations in the functional interactions between prefrontal regions (e.g., dorsolateral and medial prefrontal cortex) and medial temporal structures (e.g., hippocampus, amygdala), consequently affecting memory- and emotion-related systems [41,42]. These alterations may be associated with difficulties in memory encoding and retrieval (e.g., [39]), as well as impairments in executive functions involved in attention regulation and emotional control (e.g., [26,41]). However, not all sleep-related difficulties showed consistent associations with academic achievement. This appears to be particularly true for daytime sleepiness. Indeed, more studies included in the present review reported no significant association between daytime sleepiness and academic achievement among first-year students (e.g., [13,43,64]), contrasting with conclusions from previous reviews [47,48]. Although the ESS was the most commonly used measure of daytime sleepiness, longitudinal studies tended to report null findings [13,43], whereas cross-sectional studies yielded more heterogeneous results [29,64]. This pattern further suggests that methodological differences, including the timing of assessments, may contribute to variability in findings.

Previous reviews have indicated that university students with a morning circadian preference tend to achieve better academic outcomes [46,48]. A similar pattern was observed in one of the studies included in the present review, which found that morning chronotypes exhibited higher academic achievement than evening chronotypes [63]. Moreover, several studies reported that an evening chronotype was associated with poorer academic performance and adjustment [13,62,65], a finding that is consistent with the meta-analysis conducted by Tonetti et al. [72], which identified the same pattern among both university and younger school students. Fitzsimmons et al. [13] further reported that evening preference was associated with academic achievement among first-year students but not among second-year students. One potential explanation for these findings is social jetlag, a mismatch between an individual’s biological rhythms and the social demands imposed by daily schedules, which in the university context are largely shaped by academic activities and routines [63,65]. Given that classes and other academic activities are generally scheduled in ways that align more closely with morning than evening chronotypes, students with a morning preference may be more attentive and engaged during learning activities and better able to capitalize on daytime hours for studying and other academic tasks [63]. Conversely, students with an evening preference may face greater conflicts between academic responsibilities and social activities, which often occur during periods when they are most alert and productive. Despite growing interest in social jetlag as a factor associated with academic performance, particularly among first-year students [13], and despite evidence that a substantial proportion of university students identify themselves as evening types [19,65], the role of chronotype and circadian preference in academic achievement remains relatively underexplored. The limited number of available studies, coupled with the presence of null findings (e.g., [29]), underscores the need for further research in this area.

As discussed throughout this review, methodological diversity across studies may contribute substantially to the heterogeneity of findings. In particular, several authors have noted that much of the existing evidence relies on subjective self-report measures, with relatively few studies employing objective assessments (e.g., [46,47,48]), a pattern that was also evident in the present review. Although self-report measures offer important practical advantages, including lower costs and reduced participant burden [13,65,73], they are not without limitations. One notable concern is their susceptibility to reporting biases [74]. For example, self-reported sleep duration tends to overestimate actual sleep duration [26]. At the same time, individuals who perceive themselves as sleeping poorly, despite not differing from good sleepers on objective sleep measures obtained through electroencephalography (EEG), tend to underestimate their sleep duration to a greater extent [75]. Furthermore, when academic achievement is self-reported, the strength of the association between achievement and sleep-related variables, such as circadian preference, may be affected [72]. The incorporation of objective sleep measures, including actigraphy and wearable technologies as more accessible and cheaper solutions for studying larger samples, and polysomnography as the gold standard [76], has long been advocated as an important step forward in this field (e.g., [7,46,48,65,66]). It is not a question of prioritizing one type of measure over the other, especially given that studies investigating the agreement between subjective sleep assessments and objective actigraphy-derived sleep measures (e.g., sleep efficiency, duration, latency) have consistently shown low levels of convergence (e.g., [73,76,77]). Instead, both approaches should be used complementarily, alongside diverse sleep indicators, to provide a more comprehensive characterization of the multiple dimensions and experiences of sleep.

Likewise, the diversity of university student populations has been identified as a key consideration when examining the relationship between sleep and academic achievement (e.g., [65,66,78]). Even though the majority of studies encompassed students from different academic disciplines, five studies were conducted exclusively with medical students, a group that has been identified as particularly vulnerable to sleep disturbances compared with students from other fields and even the general population due to factors such as greater academic demands and workload [79,80]. Nonetheless, even within this subgroup of studies, the heterogeneity of findings observed in the present review persisted across different sleep domains, including sleep duration [26,27,29], sleep quality [29,64], and chronotype [29,63], underscoring the need to broaden the range of factors considered when examining the relationship between sleep and academic achievement.

In this regard, culture-related differences have been scarcely explored in the literature investigating this association [81]. This gap is particularly relevant, as existing evidence suggests that sleep duration and sleep disturbances vary across countries and geographical regions, with different factors (e.g., bedtime routines, psychological functioning) exerting varying influences depending on the sociocultural context [81,82]. Some findings even indicate that individuals from cultures characterized by shorter sleep durations do not necessarily experience adverse health outcomes, as conformity with cultural norms regarding what is considered a healthy sleep duration appears to play an important role in perceived health [82]. Although most studies included in the present review were conducted in high-income and WEIRD countries and were geographically concentrated in North America and, to a lesser extent, Western Europe and South Asia, important regions such as Africa, East Asia, the Middle East and North Africa, Eastern Europe, Central Asia, and large parts of Latin America and Southeast Asia remain underrepresented or entirely absent. This geographical imbalance limits our understanding of how sociocultural factors shape sleep across diverse cultural contexts and restricts the generalizability of existing findings to global populations. Following the recommendations of Ou et al. [82], future research could explore how cultural norms, practices, and beliefs are associated with sleep-related outcomes across different populations. University students represent a particularly valuable population for such investigations.

Relatedly, some authors have advocated for a more person-centered approach that considers how individual characteristics and psychosocial profiles interact over time [66]. This recommendation dovetails with the need to broaden the scope of future research by including more diverse and underrepresented student populations, not only at the institutional level (e.g., international students, mature students, students with specific educational needs, and first-generation students) but also across different sociocultural contexts. Therefore, alongside addressing methodological variability, future research should seek to clarify how sociocultural, health-related, and psychosocial factors shape the relationship between sleep and academic achievement.

In addition to examining whether sleep and academic achievement are associated, several studies explored whether these associations remained after accounting for a range of sociodemographic (e.g., gender, age, race/ethnicity, first-generation student status), health and lifestyle (e.g., diet, substance use, screen time), academic (e.g., STEM major, course load), and psychosocial factors (e.g., perceived stress and anxiety). Overall, the evidence suggests that associations involving sleep duration (e.g., [13,14,22,26]), sleep quality (e.g., [13]), sleep-related problems (e.g., [7,57,62]), and chronotype (e.g., [13]) generally persist after accounting for these factors.

Notably, a subset of studies also investigated factors that may influence or help explain the relationship between sleep and academic achievement (e.g., [13,43,61,62]). Affective processes have received particular attention in research on sleep quality, with evidence indicating that lower levels of perceived sleep quality are associated with poorer psychological well-being, including higher levels of emotional distress and negative affect, as well as lower levels of positive affect, life satisfaction, and optimism (e.g., [77]). Among university students, evidence also suggests that negative affective states, including depressive symptoms, may represent one of the mechanisms linking poor sleep quality to lower academic achievement. For example, Fitzsimmons et al. [13] reported that students with higher levels of depressive symptoms appeared to experience more pronounced negative associations between poor sleep quality and academic achievement. Similarly, Flueckiger et al. [61] suggested that the role of negative affect may depend on the temporal frame under consideration. While no effect was observed when sleep quality and achievement were examined globally over time, day-to-day analyses indicated that poorer sleep quality was associated with increased negative affect, which, in turn, was related to lower achievement of daily learning goals. The role of self-regulatory capacities has also been explored in the context of sleep-related difficulties. Evidence indicates that students reporting lower levels of self-control, self-regulation, and self-efficacy tend to exhibit stronger associations between sleep problems and poorer academic outcomes [43,62]. These findings are consistent with the theoretical framework proposed by Bermudez et al. [14], which suggests that sleep difficulties may affect executive functioning and related cognitive and affective processes involved in behavioral regulation. As such, self-regulatory remain a promising avenue for future research seeking to clarify the complex relationships between sleep and academic performance.

Taken together, the findings of this review underscore the importance of continuing to investigate how different factors shape the relationship between sleep and academic achievement over time, particularly across distinct stages of university adaptation and progression (e.g., [66,83]). For example, Fitzsimmons et al. [13], who followed students throughout their first two years of university, found that poorer sleep quality, shorter sleep duration, an evening chronotype, and greater social jetlag were each associated with academic achievement at the end of the first year. However, by the end of the second year, only sleep quality remained significantly associated with academic achievement. These findings suggest that the relative importance of specific sleep-related factors may vary across different stages of students’ academic trajectories. In addition to enhancing methodological consistency across studies, future research would benefit from the broader incorporation of objective sleep measures, as previously advocated by several authors (e.g., [7,46,48,65,66]). Combining quantitative and qualitative approaches may also prove valuable, as such designs could provide a deeper understanding of how first-year students perceive the role of sleep in their academic success, as well as their needs and preferences regarding support services and intervention strategies [50].

Despite the future research directions highlighted in response to limitations identified in the literature, the present review also has limitations that should be acknowledged. The search, screening, and reporting processes were performed by a single reviewer, which may have increased the potential for selection and reporting bias. Furthermore, although the search strategy did not exclude the grey literature, no eligible records from the grey literature sources were included in the review. Therefore, future reviews could consider searching the additional grey literature databases and repositories to increase the likelihood of identifying and incorporating a broader range of relevant evidence. Furthermore, as the primary aim of this scoping review was to map the available evidence, the findings should be interpreted with caution in light of the varying methodological quality of the included studies.

From a practical perspective, the implementation of sleep-friendly policies may represent a valuable strategy for supporting student success. Such initiatives could include greater flexibility in the scheduling of classes, assessments, and assignments [72]. Although these adaptations may present logistical and resource-related challenges, more targeted approaches could be considered, particularly for first-year students and courses consistently associated with lower academic performance. For example, Estevan et al. [60] examined the effects of different examination schedules and found that students who completed a midterm assessment during later shifts outperformed those assigned to earlier shifts. Importantly, this difference appeared to be associated with longer sleep duration rather than differences in study time before the examination. Another example of sleep-friendly policy involves integrating sleep-related indicators into student health surveillance systems, transition programs, and university well-being services. Likewise, sleep education initiatives incorporating behavioral and environmental recommendations to promote sleep hygiene (e.g., maintaining consistent sleep schedules, limiting caffeine and alcohol consumption before bedtime, reducing the use of sleep-disruptive technologies, reserving sleep-related spaces primarily for sleep) have been highlighted as relevant strategies (e.g., [4,26,64,84]). According to a review by Lubas and Szklo-Coxe [85], sleep education interventions for undergraduate students are generally flexible, as they can be delivered in different formats (e.g., in person or online), employ a variety of strategies (e.g., educational components, self-monitoring), and vary in duration (e.g., single-session interventions, semester-long courses). These approaches have shown effectiveness in addressing subclinical sleep-related disturbances. Their adaptability makes them feasible options for higher education institutions with varying resources, barriers, and strategic priorities, which may also change over time. Furthermore, such initiatives may be easier to integrate into programs already implemented at the high school level [9], while adapting their content to the specific demands and routines of university life [50]. It is important to note, however, that the long-term effects of these interventions on sleep-related outcomes remain insufficiently understood [85]. Similarly, their potential impact on academic achievement has received limited attention. Future research should therefore explore these issues further, as they represent a key point of debate regarding the extent to which sleep-related interventions can contribute to student retention, reduce dropout risk, and ultimately support graduation outcomes.

## 4. Conclusions

Despite the importance of academic achievement for student retention and long-term success and the growing recognition of sleep as a modifiable factor associated with academic performance, the present scoping review shows that the literature examining the relationship between sleep and academic achievement among first-year university students remains methodologically heterogeneous, with substantial variation in study designs, measurement approaches, and sleep-related indicators. The available evidence highlights sleep as a relevant factor in the transition to university, although the consistency of associations with academic achievement appears to vary across sleep domains. While findings regarding sleep duration and sleep quality are more heterogeneous, sleep problems (e.g., chronic sleep deprivation, risk of sleep disorders) and an evening chronotype are more consistently associated with less favorable academic outcomes. At the same time, the findings underscore the complexity of the relationship between sleep and academic achievement, which appears to be shaped by a range of sociocultural, health-related, psychological, and academic factors. Future research would benefit from greater methodological consistency, increased use of longitudinal designs and objective sleep measures, and a broader examination of the neurocognitive, affective, and social mechanisms underlying these associations. Particular attention should also be given to the role of individual differences in shaping distinct patterns of adaptation and academic trajectories. Overall, this review supports the consideration of sleep as an important component of initiatives aimed at promoting student success during the first year of university.

## Figures and Tables

**Table 1:** Main characteristics of the studies included in the scoping review.

Study (Country)	Study Design	Participants (nf, Age)	Sleep Measures	Academic Achievement Measures	Main Findings
Ayriza et al. [56] (Indonesia)	Cross-sectional	231 first-year students (n/a, 17–23 yo)	PSQI	First-semester GPA	X association between sleep quality and academic achievement X sleep quality did not mediate the relationship between well-being and academic achievement
Bermudez et al. [14] (Southern USA)	Cross-sectional (two academic years)	6002 first-year students, 49.3% from STEM majors (nf = 3721, Mage = 18.57)	Typical weekday sleep duration during the first 3–5 weeks of college: short sleep (<7 h), normal sleep (7–9 h), long sleep (>9 h)	Semester GPA	Short sleep (<7 h) and long sleep (>9 h) predicted worse academic achievement, even after controlling for demographics (e.g., first-generation, gender, age, race/ethnicity, employment, off-campus living), high school academic index, and psychosocial variables (e.g., stress, belonging, financial burden)
Bijvank et al. [43] (The Netherlands)	Longitudinal (beginning and end of the academic year for students starting their studies between 2010 and 2013)	1760 first-year students—Applied Sciences (nf = 1211, Mage = 19.3)	Sleeping problems—three self-report items: “I regularly have trouble falling asleep”; “I often wake up at night and have trouble falling asleep again”; “I often wake up early and have trouble falling asleep again” (1 = totally disagree, 5 = totally agree; mean score) Daytime sleepiness—two self-report items: “I feel sleepy during the first hours at school”; “I have trouble waking up in the morning, and when the alarm clock rings, I have trouble getting up” (1 = totally disagree, 5 = totally agree; mean score)	First-year dropout (yes, no) Total number of credits earned during the first examination period, according to the European Credit Transfer and Accumulation System (ECTS)	Students who reported more sleeping problems at the beginning of the first year were at a higher risk of dropping out and obtaining fewer study credits at the end of the first year The association between study credits and sleeping problems is especially evident among students who report lower self-control and self-regulation X for the analysis considering daytime sleepiness
Chen & Chen [57] (USA)	Longitudinal (two moments: spring of freshman year, spring of senior year)	3549 first-year students (nf = 2236, Mage = 18.05)	Self-report question: “How often do you feel that you are ‘sleep deprived’ (i.e., don’t get enough sleep to function effectively)?”; the reported frequency (never, seldom, occasionally, frequently, and almost always) was coded into a dichotomous variable of chronic sleep deprivation	End-of-semester GPA Graduation (within four years)	Chronic sleep deprivation was associated with lower GPA, even after controlling for demographic (e.g., age, gender, race/ethnicity, highest parental education, family income), college experience (e.g., grant/scholarship recipient, off-campus work per week, time spent on studying per week), and health and lifestyle behaviors (e.g., self-rated health, smoking, binge drinking, frequency of physical activity per week, purpose in life) Chronic sleep deprivation was associated with lower odds of college graduation (especially during the senior year)
Creswell et al. [22] (USA)	Longitudinal (early-term sleep and spring GPA)	634 first-year students from five different cohorts (n/a, n/a)	Early-term sleep characteristics assessed via wrist actigraphy: total nightly sleep time	End-of-semester GPA	Less early-term sleep time was associated with a lower end-of-semester GPA, even after controlling for other factors (e.g., gender, race, first-generation college student status, daytime sleep, academic load) Less than 6 h of nightly sleep was particularly deleterious for end-of-semester GPA relative to previous-term GPA
Deliens et al. [12] (Belgium)	Cross-sectional	101 first-year students (nf = 77, Mage = 18)	Sleeping habits questions from HBS: hours of sleep on weekdays (hours/day); sleep on weekend days (hours/day)	End-of-academic-year GPA (students who passed, failed, or did not receive a final GPA)	X difference in the amount of sleep between students who passed or failed X association between amount of sleep and academic achievement
Douris et al. [58] (USA)	Longitudinal (two moments: beginning and end of the first academic school year)	37 first-year students enrolled in a Physical Therapy program (nf = 17, Mage = 23.5)	PSQI Open-ended question of the PSQI as a measure of hours of sleep: “During the past month, how many actual hours of sleep did you get at night?”	GPA	A greater number of hours of sleep was associated with higher chances of achieving a higher GPA (≥3.5) A greater global sleep score (PSQI), meaning poorer quality of sleep, was also associated with higher chances of achieving a higher GPA (≥3.5) The authors stated that while less sleep affected academic success, lower quality of sleep did not
Driller et al. [59] (New Zealand)	Cross-sectional (two academic years)	193 first-year students enrolled in a Health, Sport and Human Performance degree (nf = 102, Mage = 19.3)	PSQI	Average of the final grades across three core subjects (first semester)	Going to bed earlier (bedtime) was associated with higher academic grades X association between other sleep quantity and quality measures (sleep efficiency, total sleep time, PSQI global score, bedtime) and academic performance X in academic performance when comparing three groups derived from the PSQI (students with poor, moderate, good sleep quality)
Estevan et al. [60] (Uruguay)	Cross-sectional (survey 1)	97 first-year students—School of Science (nf = 63, Mage = 21)	Questionnaire assessing sleep patterns (bedtime, sleep latency, sleep end) on regular nights and on the night before an examination	Correct answer rate on a midterm examination (contributing 1/3 of the final grade)	A one-hour increase in regular sleep duration was associated with a 10.8% increase in the likelihood of correct answers A one-hour increase in regular sleep duration before the test was associated with a 15.0% increase in the likelihood of correct answers 8 h-sleepers (vs. all-nighters) obtained a passing grade
	Cross-sectional (survey 2)	252 first-year students—School of Psychology (nf = 220, Mage = 23.8) divided into four shifts: 8:00 to 9:15 (n = 71); 9:45 to 11:00 (n = 71); 11:30 to 12:45 (n = 62); 13:15 to 14:30 (n = 68)	Same questionnaire as survey 1 + one additional question: “How many hours do you spend studying, adding up yesterday and today’s hours previous to the test?”	Correct answer rate on a midterm examination (contributing 1/2 of the final grade)	X regular sleep duration did not predict test performance A one-hour increase in regular sleep duration before the test was associated with a 3.8% increase in the likelihood of correct answers Relative to the 8:00 shift, the probability of achieving better test performance was higher in each of the three later shifts (i.e., test performance improved as tests’ start times were delayed) X association between the number of hours spent studying in the day/night before the test and test performance 8 h-sleepers (vs. all-nighters) obtained a passing grade
Fitzsimmons et al. [13] (USA)	Longitudinal (first and second year)	499 first-year students enrolled in a STEM major (nf = 353, Mage = 18.27)	PSQI Social jetlag: sleep schedules separated by weekdays and weekends (bedtimes, wake times, sleep durations) ESS MEQ	Cumulative GPA (end of year 1 and 2—Spring semester) Prior academic achievement: self-reported ACT scores Institutional withdrawals	Worse global sleep quality, shorter sleep durations, an evening chronotype, and greater social jetlag each independently predicted GPA at the end of the first year, even after adjusting for sociodemographic variables (sex, age, race/ethnicity), mental health indicators (depressive symptoms, anxiety), and fluid intelligence When considering GPA at the end of the second year, only global sleep quality remained a significant predictor Depressive symptoms moderated the relationship between global sleep quality and first-year GPA: the negative impact of poor sleep on academic performance was stronger among students reporting higher levels of depressive symptoms X daytime sleepiness did not predict academic achievement X links between sleep variables and institutional withdrawals in years one and two
Flueckiger et al. [61] (Switzerland)	Longitudinal (46 consecutive days over the year-end examination period from May to July)	72 first-year psychology students (nf = 51, MeAge = 21)	Daily sleep quality report using the sleep quality item of the PSQI: rate sleep quality during the previous night	Self-reported learning goal achievement: participants rated the extent to which they had achieved the learning goals set for the previous 24 h using a 5-point Likert scale (0 = not at all; 4 = completely) Grades from six final examinations, combined into a dichotomous outcome (pass vs. fail)	Students who experienced higher average sleep quality reported greater learning goal attainment On days when sleep quality was higher, participants reported better learning goal achievement X overall negative affect did not mediate the relation between sleep quality and learning goal achievement, while positive affect partially mediated this relation On days with higher sleep quality, participants reported increased positive affect and reduced negative affect, which in turn contributed to better learning goal achievement
Gaultney [62] (USA)	Longitudinal (three consecutive academic years: 2012–2014)	900 freshmen students (nf = 597, Mage = 18.58)	Sleep-50 (outcome dichotomized into risk of having at least one of four sleep disorders) Sleep hygiene questions from the Sleep-50 averaged to assess sleep quality Additional self-report questions: chronotype (preference for evening, morning, both equally, or neither); typical weekday and weekend sleep duration; sleep quality—“How restful or refreshing is your sleep?” (1 = poor, 5 = excellent) Measures were collected during the first year of university	GPA (for each of the three academic years)	Lower GPA was associated with an evening preference, being at risk for a sleep disorder, and reporting more maladaptive sleep hygiene In the absence of covariates (e.g., age, gender, ethnicity, overall health, depression, evening preference, sleep hygiene), being at risk for a sleep disorder predicted retention into the second academic year among students who remained at the university; however, this association was attenuated after adjustment for covariates After controlling for covariates, students at risk for a sleep disorder had lower GPAs for the first two years (not in the third year); among the controlled covariates, sleep hygiene and evening preference were particularly relevant By the end of the first academic year, being at risk for a sleep disorder was linked to lower GPA indirectly because it was associated with lower self-efficacy, even after accounting for depression An indirect effect was observed in the first and second year: risk for sleep disorder–depression–self-efficacy–GPA
Gupta et al. [29] (India)	Cross-sectional	167 first-year medical students (nf = 41, Mage = 18.63)	PSQI ESS MEQ Sleep diary: sleeping and waking times with related information during one week	GPA (three semester examinations)	Students with low sleep quality (PSQI ≥ 5) and excessive daytime sleepiness scores (ESS ≥ 10) showed lower mean GPA An association between mean GPA and both PSQI and ESS scores was found (the direction of the association was not specified); the association between mean GPA and PSQI was maintained after controlling for cumulative attendance X association between mean GPA and MEQ Lower mean GPA was associated with reduced mean sleep duration during the week before exams X association between GPA and sleep duration the night before the exams
Hartmann & Prichard [7] (USA)	Cross-sectional	15090 first-year students from 105 distinct schools (n/a, n/a)	Four self-report questions from the ACHA-NCHA II: “On how many of the past 7 days, did you get enough sleep so that you felt restored when you woke up in the morning?” “In the past 7 days, how often have you … awakened too early in the morning and could not get back to sleep/… felt tired, dragged out, or sleepy during the day/… have an extremely hard time falling asleep?” (mean response across these four questions, referred to as “days per week with sleep problems”)	Dropped a course or received an incomplete in the past 12 months Self-reported GPA	Students who reported more sleep disturbances during the previous week had higher rates of dropping or receiving an incomplete in a course, as well as lower cumulative GPA, holding different demographic (e.g., age, gender, race), health (e.g., difficult physical illness, diagnosed and treated depression and/or anxiety), academic (e.g., studying fulltime, having a learning disability), stress-related (e.g., number of stressors), and drug-related (e.g., binge drinking episodes, marijuana use) variables constant The negative impact of poor sleep on the likelihood of dropping a class is roughly 40% higher compared with all undergraduates Sleep problems emerged as predictors of academic achievement that were comparable to, or relatively stronger than, other health-related factors (marijuana use and binge drinking)
Manjareeka et al. [63] (India)	Cross-sectional	148 first-year medical students (nf = 80; excellent subgroup: Mage = 19.6; average subgroup: Mage = 19.9)	MCTQ (over a one-month period): morning chronotype (score < 4.28), evening chronotype (score ≥ 4.28)	Dichotomous categorization based on previous semesters’ results: excellent and average	While the excellent-performance group showed a stronger preference for the morning chronotype, the average-performance group exhibited a greater tendency toward the evening chronotype Morning chronotypes were associated with better academic achievement compared to evening chronotypes
Sahin et al. [64] (Turkey)	Cross-sectional (baseline from a sleep hygiene education intervention—Fall semester)	131 first-year medical students (nf = 70; control group—75: nf = 38, Mage = 18.1; intervention group—56: nf = 32, Mage = 18.5)	Sleep characteristics PSQI ESS	GAP (Fall semester): average score considering seven courses (0–100 points) Number of failed courses (Fall semester)	Lower PSQI scores were associated with higher GAP scores, indicating that better reported sleep quality was linked to greater academic performance X association between mean GAP and ESS X differences between number of failed courses and PSQI + ESS GAP: students with poor sleep quality (PSQI ≥ 5) < students with expected PSQI scores X differences in academic performance when ESS scores were explored
Saroha et al. [26] (India)	Longitudinal (three months)	72 first-year medical students (nf = 32, Mage = 19.1)	Daily self-report sleep diaries: total sleep duration, sleep latency, perceived quality (1 = very good, 5 = very poor)	Continuous internal assessments (average percentage)	Students who reported longer sleep duration (>6.5 h) achieved higher scores than those with moderate (5.5–6.5 h) or short (<5.5 h) sleep duration Longer sleep duration predicted better academic achievement, even after adjusting for potential confounding variables (screen time, diet, perceived stress, evening chronotype)
Sutay et al. [27] (India)	Cross-sectional	171 first-year medical students (nf = 90, n/a)	Self-report questions: sleep disorder, daytime sleep (yes, occasionally, never), duration of daytime sleep (<1 h, 1–2 h, >2 h), sleep duration prior medical school (<6 h, 6–7 h, 7–8 h, >8 h), sleep duration (<6 h, 6–7 h, 7–8 h, >8 h)	Score (percentage): prior to university entry and on the most recent examination in university	X association between sleep duration and current academic achievement Association emerged when prior academic performance was examined (the relationship was non-linear, with students sleeping 6–7 h demonstrating the highest performance)
Tavernier et al. [65] (Canada)	Longitudinal (three consecutive academic years)	942 first-year students (nf = 674, Mage = 19.01)	Perceived morningness-eveningness: ‘‘Would you describe yourself as a morning person (do your best thinking and work in the morning)?’’; ‘‘Would you describe yourself as an evening person (do your best thinking and work in the evening)?” (participants were categorized as morning-types or evening-types) Sleep problems: ISI version	SACQ	Higher levels of perceived eveningness were linked to poorer academic adjustment, increased substance use, and a greater prevalence of sleep problems Greater perceived eveningness predicted poorer academic adjustment across the first three years of university Significant effects remained after controlling for prior outcome scores, covariates (age, gender, parental education), and other predictors, while accounting for correlations among study variables
Tavernier and Willoughby [66] (Canada)	Longitudinal (three consecutive academic years)	942 first-year students (nf = 674, Mage = 19.01)	Sleep quality: ISI version Sleep–wake times for week and weekend: what time they normally fall asleep and normally wake up	Year-end academic grades (all courses)	Higher academic achievement predicted better sleep quality over time and shorter sleep duration during the week X academic achievement did not predict sleep duration during the weekend X sleep quality did not predict academic achievement over time X sleep duration during the week and on the weekend did not predict academic achievement Significant effects persisted after adjustment for prior outcome scores, covariates (age, gender, parental education), and other predictors, while accounting for correlations among study variables

Note. ACHA-NCHA = American College Health Association’s National College Health Assessment; ACT = American College Testing; ECTS = European Credit Transfer and Accumulation System; ESS = Epworth Sleepiness Scale; GAP = Global Academic Performance; GPA = Grade Point Average; HBS = Health and Behavior Survey; ISI = Insomnia Severity Index; MCTQ = Munich Chronotype Questionnaire; MEQ = Morningness-Eveningness Questionnaire; n/a = Not available/applicable; PSQI = Pittsburg Sleep Quality Index; SACQ = Student Adaptation to College Questionnaire; STEM = Science, Technology, Engineering, and Mathematics; X = No statistically significant effect.

## Data Availability

No new data were created or analyzed in this study. Data sharing is not applicable to this article.

## Supplementary Materials

The following supporting information can be downloaded at https://www.mdpi.com/article/10.3390/clockssleep8030050/s1.

## Abbreviations

The following abbreviations are used in this manuscript:

ACHA-NCHA	American College Health Association’s National College Health Assessment
ACT	American College Testing
EEG	Electroencephalography
ESS	Epworth Sleepiness Scale
GAP	Global Academic Performance
GPA	Grade Point Average
HBS	Health and Behavior Survey
ISI	Insomnia Severity Index
MCTQ	Munich Chronotype Questionnaire
MEQ	Morningness-Eveningness Questionnaire
PRISMA	Preferred Reporting Items for Systematic Reviews and Meta-Analyses
PSQI	Pittsburgh Sleep Quality Index
SACQ	Student Adaptation to College Questionnaire
STEM	Science, Technology, Engineering, and Mathematics
WEIRD	Western, Educated, Industrialized, Rich, and Democratic

## References

[1] Conley C.S., Kirsch A.C., Dickson D.A., Bryant F.B. (2014). Negotiating the Transition to College: Developmental Trajectories and Gender Differences in Psychological Functioning, Cognitive-Affective Strategies, and Social Well-Being. Emerg. Adulthood.

[2] Dias D., Sá M.J. (2014). The Impact of the Transition to HE: Emotions, Feelings and Sensations. Eur. J. Educ..

[3] Worsley J.D., Harrison P., Corcoran R. (2021). Bridging the Gap: Exploring the Unique Transition From Home, School or College Into University. Front. Public Health.

[4] Zhang K.C. (2025). “How Do I Start Strong?”: Exploring the Subjective Well-Being, Beliefs, and Lifestyles of First-Year University Students in the UK. Societies.

[5] Van Der Zanden P.J.A.C., Denessen E., Cillessen A.H.N., Meijer P.C. (2019). Patterns of Success: First-Year Student Success in Multiple Domains. Stud. High. Educ..

[6] Sosu E.M., Pheunpha P. (2019). Trajectory of University Dropout: Investigating the Cumulative Effect of Academic Vulnerability and Proximity to Family Support. Front. Educ..

[7] Hartmann M.E., Prichard J.R. (2018). Calculating the Contribution of Sleep Problems to Undergraduates’ Academic Success. Sleep Health.

[8] Westrick P.A., Schmidt F.L., Le H., Robbins S.B., Radunzel J.M.R. (2021). The Road to Retention Passes through First Year Academic Performance: A Meta-Analytic Path Analysis of Academic Performance and Persistence. Educ. Assess..

[9] Willoughby T., Dykstra V.W., Heffer T., Braccio J., Shahid H. (2023). A Long-Term Study of What Best Predicts Graduating From University Versus Leaving Prior to Graduation. J. Coll. Stud. Retent. Res. Theory Pract..

[10] Van Der Zanden P.J.A.C., Denessen E., Cillessen A.H.N., Meijer P.C. (2018). Domains and Predictors of First-Year Student Success: A Systematic Review. Educ. Res. Rev..

[11] Buková A., Tomková P., Uher I., Kimáková T., Vojtaško Ľ., Salonna F. (2024). Selected Lifestyle Factors as Students Transition from Secondary School to University in Slovakia. Front. Public Health.

[12] Deliens T., Clarys P., De Bourdeaudhuij I., Deforche B. (2013). Weight, Socio-Demographics, and Health Behaviour Related Correlates of Academic Performance in First Year University Students. Nutr. J..

[13] Fitzsimmons C.L., Carter J.R., Scullin M.K. (2026). Sleep and Circadian Predictors of Academic Performance and Retention in STEM Pathways: A Longitudinal Study in University Freshmen. Adv. Physiol. Educ..

[14] Bermudez V.N., Fearon-Drake D., Wheelis M., Cohenour M., Suntai Z., Scullin M.K. (2022). Sleep Disparities in the First Month of College: Implications for Academic Achievement. SLEEP Adv..

[15] Cheng S.H., Shih C.-C., Lee I.H., Hou Y.-W., Chen K.C., Chen K.-T., Yang Y.K., Yang Y.C. (2012). A Study on the Sleep Quality of Incoming University Students. Psychiatry Res..

[16] Supartini A., Honda T., Basri N.A., Haeuchi Y., Chen S., Ichimiya A., Kumagai S. (2016). The Impact of Sleep Timing, Sleep Duration, and Sleep Quality on Depressive Symptoms and Suicidal Ideation amongst Japanese Freshmen: The EQUSITE Study. Sleep Disord..

[17] Gellis L.A., Park A., Stotsky M.T., Taylor D.J. (2014). Associations Between Sleep Hygiene and Insomnia Severity in College Students: Cross-Sectional and Prospective Analyses. Behav. Ther..

[18] Brown F.C., Buboltz W.C. (2002). Applying Sleep Research to University Students: Recommendations for Developing a Student Sleep Education Program. J. Coll. Stud. Dev..

[19] Gaultney J.F. (2010). The Prevalence of Sleep Disorders in College Students: Impact on Academic Performance. J. Am. Coll. Health.

[20] Lund H.G., Reider B.D., Whiting A.B., Prichard J.R. (2010). Sleep Patterns and Predictors of Disturbed Sleep in a Large Population of College Students. J. Adolesc. Health.

[21] Rea E.M., DeCarlo Santiago C., Nicholson L., Heard Egbert A., Bohnert A.M. (2023). Sleep, Affect, and Emotion Reactivity in First-Year College Students: A Daily Diary Study. Int. J. Behav. Med..

[22] Creswell J.D., Tumminia M.J., Price S., Sefidgar Y., Cohen S., Ren Y., Brown J., Dey A.K., Dutcher J.M., Villalba D. (2023). Nightly Sleep Duration Predicts Grade Point Average in the First Year of College. Proc. Natl. Acad. Sci. USA.

[23] Dagani J., Buizza C., Cela H., Sbravati G., Rainieri G., Ghilardi A. (2024). The Interplay of Sleep Quality, Mental Health, and Sociodemographic and Clinical Factors among Italian College Freshmen. J. Clin. Med..

[24] Hirshkowitz M., Whiton K., Albert S.M., Alessi C., Bruni O., DonCarlos L., Hazen N., Herman J., Katz E.S., Kheirandish-Gozal L. (2015). National Sleep Foundation’s Sleep Time Duration Recommendations: Methodology and Results Summary. Sleep Health.

[25] Watson N.F., Badr M.S., Belenky G., Bliwise D.L., Buxton O.M., Buysse D., Dinges D.F., Gangwisch J., Grandner M.A., Kushida C. (2015). Recommended Amount of Sleep for a Healthy Adult: A Joint Consensus Statement of the American Academy of Sleep Medicine and Sleep Research Society. J. Clin. Sleep Med..

[26] Saroha R., Singh S., Kosvi M., Ajmera N., Grover P. (2025). Impact of Sleep Deprivation on Cognition and Academic Scores: A Three-Month Longitudinal Study Among Indian Medical Students. Cureus.

[27] Sutay S.S., Sheikh N.A., Rath R.S., Vasudeva A. (2022). Sleep Patterns, Issues, Reasons for Sleep Problems, and Their Impact on Academic Performance Among First-Year Medical Students in Central India. Maedica—J. Clin. Med..

[28] Driller M., Suppiah H., Gastin P.B., Beaven C.M. (2022). Questionnaire-Derived Sleep Habits and Academic Achievement in First Year University Students. Clocks Sleep.

[29] Gupta S., Prithviraj M., Gangwar A., Rath R.S. (2023). Impact of Sleep Duration, Quality, and Chronotype on Learning and Academic Performance: A Cross-Sectional Study Among First Year Medical Students of a Tertiary Care Institute. Cureus.

[30] Kosendiak A.A., Adamczak B.B., Kuźnik Z., Makles S. (2024). Impact of Medical School on the Relationship between Nutritional Knowledge and Sleep Quality—A Longitudinal Study of Students at Wroclaw Medical University in Poland. Nutrients.

[31] Huang C.-F., Yang L.-Y., Wu L.-M., Liu Y., Chen H.-M. (2014). Determinants of Daytime Sleepiness in First-Year Nursing Students: A Questionnaire Survey. Nurse Educ. Today.

[32] King N., Pickett W., Keown-Stoneman C.D.G., Miller C.B., Li M., Duffy A. (2023). Changes in Sleep and the Prevalence of Probable Insomnia in Undergraduate University Students over the Course of the COVID-19 Pandemic: Findings from the U-Flourish Cohort Study. BJPsych Open.

[33] Li Y., Bai W., Zhu B., Duan R., Yu X., Xu W., Wang M., Hua W., Yu W., Li W. (2020). Prevalence and Correlates of Poor Sleep Quality among College Students: A Cross-Sectional Survey. Health Qual. Life Outcomes.

[34] Fernández-Mendoza J., Vela-Bueno A., Vgontzas A.N., Olavarrieta-Bernardino S., Ramos-Platón M.J., Bixler E.O., De La Cruz-Troca J.J. (2009). Nighttime Sleep and Daytime Functioning Correlates of the Insomnia Complaint in Young Adults. J. Adolesc..

[35] Garmabi M., Andishmand Z., Naderi F., Sharifnezhad A., Darrudi F., Malekzadeh R., Amini A., Gholami A. (2024). The Prevalence of Depression and Anxiety and Its Association with Sleep Quality in the First-Year Medical Science Students. Depress. Res. Treat..

[36] Hyndych A., El-Abassi R., Mader E.C. (2025). The Role of Sleep and the Effects of Sleep Loss on Cognitive, Affective, and Behavioral Processes. Cureus.

[37] Walker M.P. (2009). The Role of Sleep in Cognition and Emotion. Ann. N. Y. Acad. Sci..

[38] Heissel J.A., Levy D.J., Adam E.K. (2017). Stress, Sleep, and Performance on Standardized Tests: Understudied Pathways to the Achievement Gap. AERA Open.

[39] Newbury C.R., Crowley R., Rastle K., Tamminen J. (2021). Sleep Deprivation and Memory: Meta-Analytic Reviews of Studies on Sleep Deprivation before and after Learning. Psychol. Bull..

[40] Crowley R., Alderman E., Javadi A.-H., Tamminen J. (2024). A Systematic and Meta-Analytic Review of the Impact of Sleep Restriction on Memory Formation. Neurosci. Biobehav. Rev..

[41] Harrington M.O., Ashton J.E., Sankarasubramanian S., Anderson M.C., Cairney S.A. (2021). Losing Control: Sleep Deprivation Impairs the Suppression of Unwanted Thoughts. Clin. Psychol. Sci..

[42] Yoo S.-S., Gujar N., Hu P., Jolesz F.A., Walker M.P. (2007). The Human Emotional Brain without Sleep—A Prefrontal Amygdala Disconnect. Curr. Biol..

[43] Nije Bijvank M., Tonnaer G.H., Jolles J. (2017). Self-Perceived Problems in Sleeping and in Self-Control Are Related to First Year Study Success in Higher Education. Front. Educ..

[44] Bloomfield L.S.P., Fudolig M.I., Kim J.N., Llorin J., Lovato J., McGinnis E.W., McGinnis R.S., Price M., Ricketts T.H., Sheridan Dodds P. (2025). Predictors of Anxiety Trajectories in Cohort of First-Year College Students. JAACAP Open.

[45] Milojevich H.M., Lukowski A.F. (2016). Sleep and Mental Health in Undergraduate Students with Generally Healthy Sleep Habits. PLoS ONE.

[46] Hershner S. (2020). Sleep and Academic Performance: Measuring the Impact of Sleep. Curr. Opin. Behav. Sci..

[47] Seoane H.A., Moschetto L., Orliacq F., Orliacq J., Serrano E., Cazenave M.I., Vigo D.E., Perez-Lloret S. (2020). Sleep Disruption in Medicine Students and Its Relationship with Impaired Academic Performance: A Systematic Review and Meta-Analysis. Sleep Med. Rev..

[48] Suardiaz Muro M., Morante Ruiz M., Ortega Moreno M., Ruiz M.A., Martín Plasencia P., Vela Bueno A. (2020). Sueño y rendimiento académico en estudiantes universitarios: Revisión sistemática. Rev. Neurol..

[49] Soon C.S., Chua X.Y., Leong R.L.F., Ong J.L., Massar S.A.A., Qin S., Chong K.H.M., Onnela J.-P., Chee M.W.L. (2025). A Longitudinal Study of Sleep in University Freshmen: Facilitating and Impeding Factors. Sleep.

[50] Tadros M., Li S., Upton E., Newby J., Werner-Seidler A. (2023). Preferences of University Students for a Psychological Intervention Designed to Improve Sleep: Focus Group Study. JMIR Hum. Factors.

[51] Zhang D., Qu Y., Zhai S., Li T., Xie Y., Tao S., Zou L., Tao F., Wu X. (2023). Association between Healthy Sleep Patterns and Depressive Trajectories among College Students: A Prospective Cohort Study. BMC Psychiatry.

[52] Hadie S.N.H. (2024). ABC of a Scoping Review: A Simplified JBI Scoping Review Guideline. Educ. Med. J..

[53] Munn Z., Peters M.D.J., Stern C., Tufanaru C., McArthur A., Aromataris E. (2018). Systematic Review or Scoping Review? Guidance for Authors When Choosing between a Systematic or Scoping Review Approach. BMC Med. Res. Methodol..

[54] Page M.J., McKenzie J.E., Bossuyt P.M., Boutron I., Hoffmann T.C., Mulrow C.D., Shamseer L., Tetzlaff J.M., Akl E.A., Brennan S.E. (2021). The PRISMA 2020 Statement: An Updated Guideline for Reporting Systematic Reviews. BMJ.

[55] Tricco A.C., Lillie E., Zarin W., O’Brien K.K., Colquhoun H., Levac D., Moher D., Peters M.D.J., Horsley T., Weeks L. (2018). PRISMA Extension for Scoping Reviews (PRISMA-ScR): Checklist and Explanation. Ann. Intern. Med..

[56] Ayriza Y., Setiawati F.A., Nurhayati S.R., Gumelar S.R., Sholeha E.P.D.R. (2019). Does Sleep Quality Serve as a Mediator between Well-Being and Academic Achievement?. J. Cakrawala Pendidik..

[57] Chen W.-L., Chen J.-H. (2019). Consequences of Inadequate Sleep during the College Years: Sleep Deprivation, Grade Point Average, and College Graduation. Prev. Med..

[58] Douris P.C., Hall C.A., Jung M.-K. (2023). The Relationship between Academic Success and Sleep, Stress and Quality of Life during the First Year of Physical Therapy School. J. Am. Coll. Health.

[59] Driller M.W., Rogers T., Hébert-Losier K., Beaven C.M. (2021). Body Mass Changes and Markers of Fitness, Health, and Well-Being over the First Semester of University in New Zealand Students. J. Sci. Sport Exerc..

[60] Estevan I., Sardi R., Tejera A.C., Silva A., Tassino B. (2021). Should I Study or Should I Go (to Sleep)? The Influence of Test Schedule on the Sleep Behavior of Undergraduates and Its Association with Performance. PLoS ONE.

[61] Flueckiger L., Lieb R., Meyer A.H., Mata J. (2014). How Health Behaviors Relate to Academic Performance via Affect: An Intensive Longitudinal Study. PLoS ONE.

[62] Gaultney J.F. (2016). Risk for Sleep Disorder Measured During Students’ First College Semester May Predict Institutional Retention and Grade Point Average Over a 3-Year Period, with Indirect Effects Through Self-Efficacy. J. Coll. Stud. Retent. Res. Theory Pract..

[63] Manjareeka M., Dasgupta S., Kanungo P., Das R.C. (2025). Perceived Stress and Academic Achievement among Medical Students with Different Chronotypes: A Cross Sectional Study on First Year Medical Students from India. BMC Med. Educ..

[64] Sahin E.M., Ozturk L., Oyckein D.G., Uludag A. (2016). Effects of Sleep Hygiene Education on Subjective Sleep Quality and Academic Performance. Ann. Clin. Anal. Med..

[65] Tavernier R., Munroe M., Willoughby T. (2015). Perceived Morningness–Eveningness Predicts Academic Adjustment and Substance Use across University, but Social Jetlag Is Not to Blame. Chronobiol. Int..

[66] Tavernier R., Willoughby T. (2014). Bidirectional Associations between Sleep (Quality and Duration) and Psychosocial Functioning across the University Years. Dev. Psychol..

[67] Fergus S. (2022). Are Undergraduate Students Studying Smart? Insights into Study Strategies and Habits across a Programme of Study. J. Univ. Teach. Learn. Pract..

[68] Okur A., Keskin A., Aydın Berktaş Ö. (2026). The Relationship between Sleep Quality and Academic Achievement among Students in Health-Related Disciplines: A Cross-Sectional Study. BMC Med. Educ..

[69] Casanova J.R., Castro-López A., Bernardo A.B., Almeida L.S. (2023). The Dropout of First-Year STEM Students: Is It Worth Looking beyond Academic Achievement?. Sustainability.

[70] Chan N.Y., Wu W.J., Chan J.W.Y., Chan K.C.C., Li A.M., Chan S.S.M., Hau K.T., Wing Y.K. (2023). Sleep and Academic Performance among Students in Hong Kong: Curvilinear Relationship Suggesting an Optimal Amount of Sleep. Sleep Med..

[71] Armand M.A., Biassoni F., Corrias A. (2021). Sleep, Well-Being and Academic Performance: A Study in a Singapore Residential College. Front. Psychol..

[72] Tonetti L., Natale V., Randler C. (2015). Association between Circadian Preference and Academic Achievement: A Systematic Review and Meta-Analysis. Chronobiol. Int..

[73] Girschik J., Fritschi L., Heyworth J., Waters F. (2012). Validation of Self-Reported Sleep Against Actigraphy. J. Epidemiol..

[74] Pierson-Bartel R., Ujma P.P. (2024). Objective Sleep Quality Predicts Subjective Sleep Ratings. Sci. Rep..

[75] Masaki M., Tsumoto S., Tani A., Tominaga M., Seol J., Chiba S., Miyanishi K., Nishida K., Kawana F., Amemiya T. (2025). Discrepancies between Subjective and Objective Sleep Assessments Revealed by In-Home Electroencephalography during Real-World Sleep. Proc. Natl. Acad. Sci. USA.

[76] Aili K., Åström-Paulsson S., Stoetzer U., Svartengren M., Hillert L. (2017). Reliability of Actigraphy and Subjective Sleep Measurements in Adults: The Design of Sleep Assessments. J. Clin. Sleep Med..

[77] Jackowska M., Ronaldson A., Brown J., Steptoe A. (2016). Biological and Psychological Correlates of Self-Reported and Objective Sleep Measures. J. Psychosom. Res..

[78] Okano K., Kaczmarzyk J.R., Dave N., Gabrieli J.D.E., Grossman J.C. (2019). Sleep Quality, Duration, and Consistency Are Associated with Better Academic Performance in College Students. npj Sci. Learn..

[79] Azad M.C., Fraser K., Rumana N., Abdullah A.F., Shahana N., Hanly P.J., Turin T.C. (2015). Sleep Disturbances among Medical Students: A Global Perspective. J. Clin. Sleep Med..

[80] Blakey H., Blanshard E., Cole H., Leslie F., Sen R. (2008). Are Medical Students Socially Exclusive? A Comparison with Economics Students. Med. Educ..

[81] Jeon M., Dimitriou D., Halstead E.J. (2021). A Systematic Review on Cross-Cultural Comparative Studies of Sleep in Young Populations: The Roles of Cultural Factors. Int. J. Environ. Res. Public Health.

[82] Ou C., Lou N.M., Maheshka C., Shi M., Takemura K., Cheung B., Heine S.J. (2025). Healthy Sleep Durations Appear to Vary across Cultures. Proc. Natl. Acad. Sci. USA.

[83] Cha H.S. (2019). Influence on Adjustment of University Life among Nursing Students. J. Korea Acad.-Ind. Coop. Soc..

[84] Gardani M., Bradford D.R.R., Russell K., Allan S., Beattie L., Ellis J.G., Akram U. (2022). A Systematic Review and Meta-Analysis of Poor Sleep, Insomnia Symptoms and Stress in Undergraduate Students. Sleep Med. Rev..

[85] Lubas M.M., Szklo-Coxe M. (2019). A Critical Review of Education-Based Sleep Interventions for Undergraduate Students: Informing Future Directions in Intervention Development. Adolesc. Res. Rev..

