# Supplementary material for: Evidence on Sleep and Academic Achievement in First-Year University Students: A Scoping Review
Source: Clocks Sleep. 2026 Aug 24;8(3):50. doi: 10.3390/clockssleep8030050 (PMC13605022; doi:10.3390/clockssleep8030050)
Supplement: Supplementary file 1 [file clockssleep-08-00050-s001.zip › clockssleep-4462671-supplementary.pdf]

Supplementary Materials

Table S1. Summary of search expressions used in the literature search

Database	Query
PubMed	(((college OR university OR "higher education" OR postsecondary) AND ("first year" OR freshm*)) AND (achievement OR adjustment OR dropout OR engagement OR integration OR retention OR success)) AND (sleep)
Scopus	(TITLE-ABS-KEY (college OR university OR "higher education" OR postsecondarScy) AND TITLE-ABS-KEY ("first year" OR freshm*) AND TITLE-ABS-KEY (achievement OR adjustment OR dropout OR engagement OR integration OR retention OR success) AND TITLE-ABS-KEY (sleep))
Web of Science	(TS=(college OR university OR “higher education” OR postsecondary) AND TS=(“first year” OR freshm*) AND TS=(achievement OR adjustment OR dropout OR engagement OR integration OR retention OR success) AND TS=(sleep))
